# COVID-19 vaccine and menstrual conditions in female: data analysis of the Vaccine Adverse Event Reporting System (VAERS)

**DOI:** 10.1186/s12905-022-01934-4

**Published:** 2022-10-05

**Authors:** Bing Zhang, Xiao Yu, Jinxing Liu, Jinbao Liu, Pengfei Liu

**Affiliations:** 1 First School of Clinical Medicine, Shandong University of Traditional Chinese Medicine, Jinan, 250355 Shandong China; 2 Department of Gynecology, Affiliated Hospital of Shandong University of Traditional Chinese Medicine, Jinan, 250000 Shandong China; 3 Department of Orthopedics, Affiliated Hospital of Shandong University of Traditional Chinese Medicine, Jinan, 250000 Shandong China; 4 Department of Reproduction and Genetics, Affiliated Hospital of Shandong University of Traditional Chinese Medicine, Jinan, 250000 Shandong China

**Keywords:** VAERS, COVID-19 vaccine, Menstrual disorders events, 30–49 years, Risk signals

## Abstract

**Background:**

In reports of adverse reactions following vaccination with the coronavirus disease 2019(COVID-19) vaccines, there have been fewer reports of concern for menstrual disorders in female.

**Objective:**

Our study employed Vaccine Adverse Event Reporting System (VAERS) to investigate and analyze the relationship between COVID-19 Vaccines and menstrual disorders in female.

**Methods:**

We collected reports of menstrual disorders in VAERS from July 2, 1990 to November 12, 2021, and performed a stratified analysis. The potential relationship between COVID-19 vaccine and reports of menstrual disorders was evaluated using the Reporting Odds Ratio (ROR) method.

**Results:**

A total of 14,431 reports of menstrual disorders were included in the study, and 13,118 were associated with COVID-19 vaccine. The ROR was 7.83 (95% confidence interval [95%CI]: 7.39–8.28). The most commonly reported event was Menstruation irregular (4998 reports), and a higher percentage of female aged 30–49 years reported menstrual disorders (42.55%) after exposure to COVID-19 Vaccines. Both for all reports of menstrual disorders (ROR = 5.82; 95%CI: 4.93–6.95) and excluding reports of unknown age (ROR = 13.02; 95%CI: 10.89–15.56),suggest that female age may be associated with menstrual disorders after vaccination with the COVID-19 Vaccines.

**Conclusion:**

There is a potential safety signal when the COVID-19 vaccine is administered to young adult female (30–49 years old), resulting in menstrual disorders in. However, due to the well-known limitations of spontaneous reporting data, it is challenging to explicity classify menstrual disorders as an adverse event of the COVID-19 Vaccines, and reports of adverse reactions to COVID-19 Vaccines in this age group should continue to be tracked.

## Impacts on practice

1. After the launch of the COVID-2019 vaccine, consideration attention has been focused on special populations, such as elderly, children, pregnant women, and even congenital allergies, while less attention is paid to reports of adverse reactions in adults women after vaccination.
2. The proportion of women aged 30–49 years reporting menstrual disorders after vaccination has risen, and reports of adverse reactions in this age group in women should continue to be monitored.

## Background

Coronavirus disease of 2019 [1] (COVID-19) is a highly pathogenic viral infection caused by the severe acute respiratory syndrome coronavirus 2 (SARS‐CoV‐2). COVID-19 infection remains a global concern since the end of 2019 to date. According to WHO, as of January 6, 2022, 293,750,692 cases of COVID-19 have been diagnosed worldwide, and 55,972,114 instances have been diagnosed in the United States. For epidemic control, the safe and effective vaccine is the long-term option for conquering the COVID-19 global pandemic [2]. Research studies [3] have shown that an average vaccination level of roughly 80 doses per 100 inhabitants between countries can sustain a reduction in the number of confirmed cases and deaths. The recent emergence of the Omicron variant further emphasizes the necessity of vaccination, and prevention efforts needed to protect against COVID-19.

The U.S. Food and Drug Administration (FDA) approved three COVID-19 vaccines for emergency use in the United States in mid-December 2020 [4, 5] and late February 2021 [6], respectively. By the end of 2021, more than a dozen COVID-19 vaccines in six categories are approved for use worldwide. Local and systemic reactions were the predominant types of adverse events observed in pre-emergency authorization clinical trials of these vaccines. Because of the short development cycle of COVID-19 vaccines, there are continuing concerns regarding the post-marketing safety and efficacy of vaccines. Accordingly, in order to continue to monitor the post-marketing safety of vaccines, countries throughout the world have established different measures to gather information about vaccine recipients. The Centers for Disease Control and Prevention in the United States uses the Vaccine Adverse Reaction Reporting System (VAERS) for ongoing monitoring of the safety of the COVID-19 vaccine [7].

Since the launch of the COVID-19 vaccine, more research points have focused on special groups such as the elderly [8], adolescents [9, 10], and maternity [11, 12]. More attention is devoted to aspects such as rare allergic reactions [13] and cardiovascular system diseases [14] after vaccination, whereas less attention is paid to female's menstrual circumstances. In a cohort study [15] that included 3959 individuals (2,403 vaccinated with COVID-19 vaccine; 1,556 unvaccinated): COVID-19 vaccination is associated with a slight change in cycle length but not menses length. In a questionnaire which included 164 women [16], it was suggested that temporary and self-limiting menstrual cycle irregularities may occur in some vaccinated women, regardless of the type of COVID-19 vaccine. Henceforth, continuous attention to the effects of COVID-19 exposure on women's health and adverse effects after vaccination (especially menstrual problems) is mandatory [17].The purpose of this study is to discuss the safety of COVID-19 vaccines in female based on real data spontaneously reported in the VAERS and to attempt to analyze the potential relationship between COVID-19 vaccines and trends in reporting of menstrual disorders.

## Aim of the study

Using the US Vaccine Adverse Event Reporting System (VAERS) database, this research sought to identify indications of menstrual disorder-like adverse responses after COVID-2019 vaccination.

## Method

### Database

Established in 1990, VAERS [18] (https://vaers.hhs.gov/) is a system for spontaneous and voluntary reporting of any suspected adverse drug reactions (ADRs) associated with vaccine use, jointly administered by the Centers for Disease Control and Prevention (CDC) and the Food and Drug Administration (FDA). The primary purpose of VAERS is to detect early signals and generate hypotheses concerning possible new vaccine adverse events that were not identified in premarketing trials. VAERS accepts reports from vaccine recipients, parents, health care providers, vaccine manufacturers, and others. The VAERS report form collects information about the individual who received the vaccine, the type number of vaccine received, and the adverse reaction itself.

Signs and symptoms of an adverse reaction are coded using the Medical Dictionary of Regulatory Activities (MedDRA) [19]. A single VAERS report can contain multiple MedDRA preferred terms. The following reports are categorized as "serious" reports in compliance with the US Code of Federal Regulations [20]: Hospitalization, Prolonged hospitalization, Emergency room visits, Disability, Life-threatening, or Death.

### Dataset

After eliminating sensitive patient information, the original data files were downloaded from the VAERS website in comma-separated value (CSV) format. We retrieved the dataset in VAERS from the 1990 build through November 12, 2021, both U.S. regions and non-U.S. regions. The dataset consists of three separate data files for data, vaccines, and symptoms.

### Data selection

Use navicat15 for data mining. The following strategy was implemented: (1) Exclude all reports of male and unknown gender. Search for the following MedDRA preferred terms: "Menstruation irregular" "Menstruation delayed" "Menstrual disorder" "Hypomenorrhoea" "Menorrhagia" "Intermenstrual bleeding" "Metrorrhagia" "Amenorrhoea" (2) Each report was srutinised to clarify that the menstrual disorders were not caused by pregnancy (3) The ID, type of vaccine injected, and dose of injection were checked for each report, and reports with multiple repetitions of the same ID were excluded. (4) We extracted information on ID, vaccine type, vaccine dose, age, type of menstrual disorder, patient outcome, vaccination date, reporting interval, and past history from each report. Detailed interpretation of the reports can be found in the VAERS Data Use Guide [21]*.*

### Data analysis

The integration, extraction, and filtering of the reported data are performed by navicat15, and the description and statistical analysis of the data are realized by Free Statistics software version 1.4.

We summarized the fundamental characteristics of the reports by various categories of menstrual disorders, age, reporting interval, severity, and past history, and performed a stratified analysis of age and reporting interval. Characterization of the types of menstrual disorders reported for different vaccine types, statistical analysis using Fisher's exact test. A line graph is used to exemplify the trend in the type of report. The Reporting Odds Ratio (ROR) [22] was used to assess the disproportionate reporting of adverse reactions, with the lower limit of the ROR 95% confidence interval [95%CI] > 1 and a number of reports ≥ 3 suggesting the generation of an adverse signal. We performed the following sensitivity analyses of menstrual disorders events: (1) US and non-US regions; (2) stratification by age; and (3) exclusion of cases of unknown age.

VAERS is a self-reporting vaccine adverse reaction surveillance system that conforms to the definition of a study as specified in 45 CFR 46 [23]. Exemptions (2018Requirements). It is not subject to Institutional Review Board review and informed consent requirements.

## Results

### Reports of menstrual disorders in Vaccine Adverse Event Reporting System (VAERS)

**Fig. 1:**
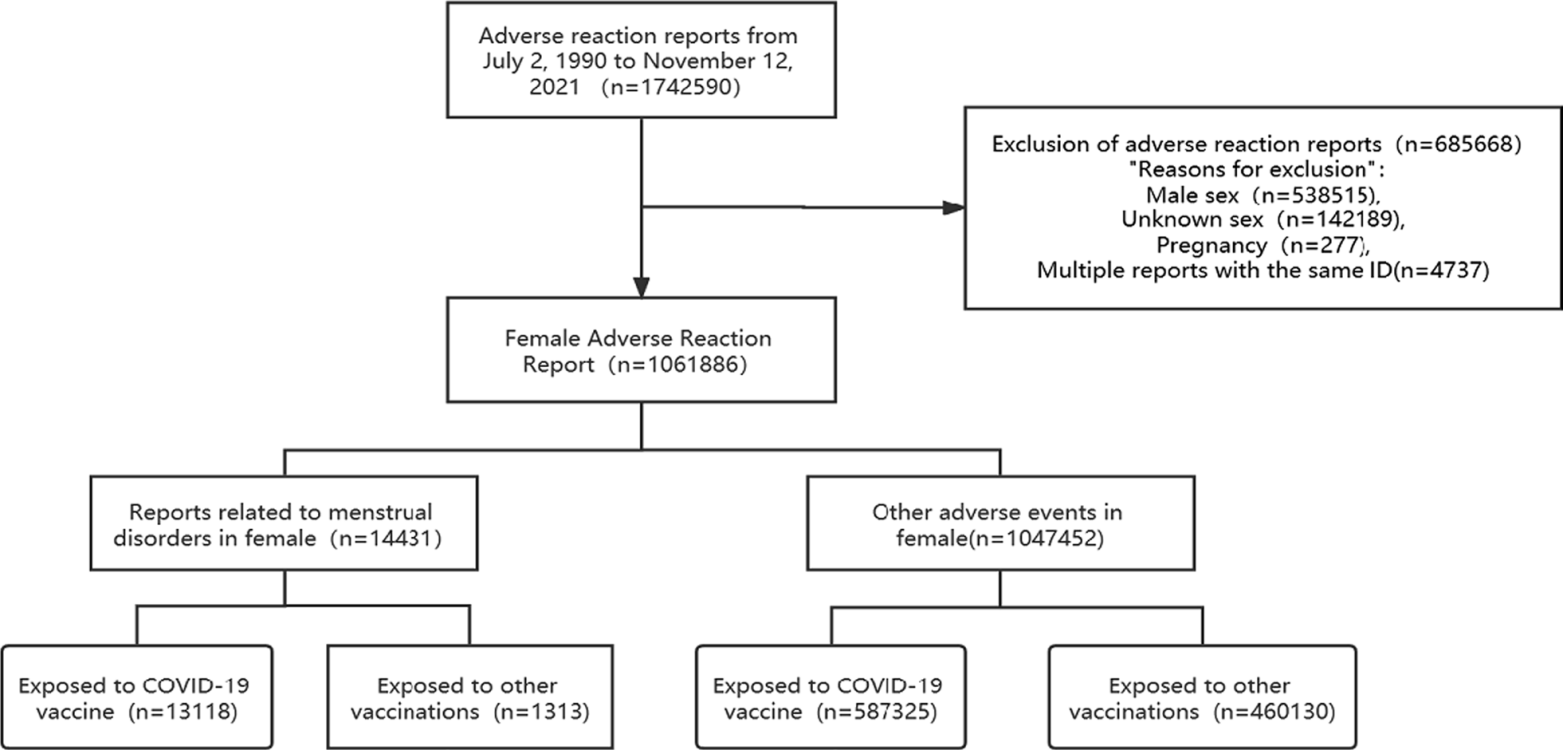
Flow diagram of case inclusion in this study

Figure 1 (Flow diagram of case inclusion in this study) depicts the report selection procedure, including the reasons for exclusion. By November 12, 2021, 1,742,590 cases of adverse events were recorded in the VAERS database, and 60.94% of them were female. The category including menstrual disorders events 14,331 reports (1.36%), of which 13,118 (90.90%) were exposed to COVID19 vaccine and 13,13 (9.10%) were exposed to another vaccine. There were 1,047,452 (98.64%) other adverse events, 587,325 (56.07%) were exposed to COVID-19 vaccine and 460,130 (43.93%) were exposed to other vaccines.

### Characterization of menstrual disorders events

Table 1 outlines the fundamental features of the 14,431 instances of menstrual disorders that have been documented. Reports of menstrual disorders are not mutually exclusive of each other, and multiple conditions may be encompassed in one adverse reaction report. The most prevalent event in both groups was Menstruation irregular, with 4626 cases (35.26%) reported in the COVID-19 vaccine group and 372 cases (28.33%) in the non-COVID-19 vaccine group. The COVID-19 vaccine group reported 2698 cases (20.57%) of Menstruation delayed,2088 cases (15.92%) of Intermenstrual bleeding, and Menorrhagia was reported only 28 cases (0.21%). The non-COVID-19 vaccine group reported 251 cases of Metrorrhagia (19.12%), 301 cases of Amenorrhoea (22.90%) and only 6 cases of Intermenstrual bleeding (0.46%).

**Table 1:** Summary of characteristics of menstrual disorders

	Total (N = 14,431)		Non COVID-19 vaccine (N = 1313)		COVID-19 vaccine (N = 13,118)	
	n	%	n	%	n	%
*Menstruation irregular event types*						
Menstruation irregular	4998	34.63	372	28.33	4626	35.26
Menstruation delayed	2803	19.42	105	8.00	2698	20.57
Menstrual disorder	2088	14.47	127	9.67	1962	14.96
Metrorrhagia	1922	13.32	251	19.12	1671	12.74
Amenorrhoea,	1956	13.55	301	22.92	1655	12.62
Menorrhagia	260	1.80	232	17.67	28	0.21
Hypomenorrhoea	1076	7.46	33	2.51	1043	7.95
Intermenstrual bleeding	2094	14.51	6	0.46	2088	15.92
Age (years)*						
Median	35	(27.0, 42.0)	16	(13.0, 24.0)	36	(30.0,43.0)
< 20	926	6.46	641	48.82	285	2.17
20 ~ 29	1952	13.62	211	16.07	1741	13.27
30 ~ 39	3255	22.71	79	6.02	3176	24.21
40 ~ 49	2476	17.28	70	5.33	2406	18.34
≥ 50	568	3.96	8	0.61	560	4.27
Unknow	5254	36.66	304	23.15	4950	37.73
Time between reports(days)**						
Median	3	(1.0, 15.0)	8	(0.0, 49.8)	3	(1.0, 14.0)
< 100	11,463	79.43	586	44.63	10,877	82.92
100 ~ 200	123	0.85	39	2.97	84	0.64
200 ~ 300	22	0.15	21	1.60	1	0.01
≥ 300	73	0.51	60	4.57	13	0.10
Unknow	2750	19.06	607	46.23	2143	16.34
*Serious adverse event*						
Death	3		3	0.23	0	
Life-threatening	126	0.87	45	3.43	81	0.62
Emergency room visits	406	2.81	406	30.92	0	
Hospitalized	540	3.74	244	18.58	296	2.26
Prolonged hospitalization	28	0.19	14	1.07	14	0.11
Disability	877	6.08	189	14.39	688	5.24
*History and current*						
Taking medications	5526	38.56	273	20.79	5253	40.04
Current Diseases	1175	8.20	191	14.55	984	7.50
Past Diseases	6481	45.22	277	21.10	6204	47.29

Multiple MedRDA terms can exist in one adverse reaction report, so the sum of the individual events of menstrual disorders events may be greater than the total number of reports

Fisher’s exact test: α = 0.05,

*P value < 0.001,

**P value < 0.001

The median age at the time of reporting was 35 years in both groups, with a mean age of 36 years in the COVID-19 vaccine group, which was greater than 16 years in the non-COVID-19 vaccine group. A high proportion of the reported age was undetermined in both groups. Nearly half (48.82%) of the reported menstrual irregularities in the non-COVID-19 vaccine group were reported in the younger age group (< 20 years). Whereas in the COVID-19 vaccine group, a higher proportion (42.55%) was reported in the prime age group (30–49 years). After Fisher's exact test, there was a discrepancy between the two age groups (*P* value < 0.001).

The interval from vaccine exposure to reported onset was reported in 11,681 cases (80.94%), with a median of 3.0 days. There were 10,877 cases (82.92%) in the COVID-19 vaccine group with an adverse reaction reporting interval < 100 days. The non-COVID-19 vaccination group had an average reporting gap of 8 days. After Fisher's exact test, there was a difference in the reporting interval between the two groups (*P* value < 0.001). The following 20 non-COVID-19 vaccinations have been linked to recorded cases of menstrual disorders: Influenza virus vaccine(38 reports), Hepatitis B virus vaccine(51reports), Tetanus and diphtheria toxoids vaccine(9 reports), Pneumococcal vaccine (3 reports), Varivax-varicella virus live(14reports), Tetanus toxoid (1report), Human papillomavirus(1073 reports), Hepatitis A (12 reports), Anthrax vaccine (20 reports), Measles(1report), Measles, mumps and rubella virus vaccine(15 reports), Lyme disease vaccine(4reports), Rabies virus vaccine(2reports), Smallpox vaccine(2reports), Meningococcal conjugate vaccine(3 reports), Hepatitis A + hepatitis B vaccine(4 reports), Ebola Zaire vaccine(1 report), Meningococcal group b vaccine(1 report), Varicella-zoster vaccine(1 report), Unknow(57 reports).

The reported species of serious adverse events were mainly related to Death, Life-threatening, Emergency room visits, Hospitalized, Prolonged hospitalization, and Disability. There were no reports of deaths in the COVID-19 vaccine group, and a total of 1079 serious adverse events were reported (8.22%). 901 serious adverse events (68.62%) were documented in the non- COVID-19 vaccine group, three fatalities were reported which were the result of exposure to Human papillomavirus vaccine (2 reports) and Hepatitis B virus vaccine (1 report). More than one-third of the reports in both groups mentioned a prescription or nonprescription drugs that the vaccine recipient was taking at the time of vaccination and 1175 cases (8.20%) were suffering from a disease, while 6481 cases (45.22%) had been diagnosed with a disease prior to vaccination.

### Characterization of different COVID-19 vaccine types

Table 2 describes the characteristics of the 13,118 menstrual disorders reported as a consequence of exposure to the COVID-19 vaccine. 9613 cases (73.28%) were reported in relation to Pfizer-Biontech, 2748 cases (20.95%) for Moderna and 742 cases (5.66%) for Janssen. The reported rates of other menstrual events differed between groups (*p* < 0.001), except Intermenstrual bleeding, Hypomenorrhoea, Menorrhagia. Comparison between groups revealed that the distribution of reports of menstrual disorders by age group was heterogeneous (*p* < 0.001). Except for the type of vaccine that could not be characterized, the remaining three groups reported significantly higher proportions in the 30–39 age group than in other age groups, respectively accounting for 19.53%, 38.54%, and 31.67% of the total. The dose distribution by injected vaccine was likewise heterogeneous (*P* < 0.001), with Dose 1 being reported at a significantly higher rate than Dose 2 and Dose 3. Only 1596 cases (16.60%) of vaccine recipients recovered from the adverse event when the adverse reaction information was reported, and 66.33% did unrecoverable at the time of reporting.

**Table 2:** Characterization of different COVID-19 vaccine types

	Pfizer-Biontech (n = 9613)		Moderna (n = 2748)		Janssen (n = 742)		Unknown (n = 15)	
	n	%	n	%	n	%	n	%
*Menstruation irregular event types*								
Menstruation irregular	3327	34.61	991	36.06	302	40.70	6	40.00
Menstruation delayed	2152	22.39	454	16.52	88	11.86	4	26.67
Menstrual disorder	1141	11.87	614	22.34	204	27.49	2	13.33
Metrorrhagia	1444	15.02	199	7.24	27	3.64	1	6.67
Amenorrhoea	1094	11.38	453	16.48	105	14.15	3	20.00
Menorrhagia	26	0.27	2	0.07	0	0.00	0	0.00
Hypomenorrhoea	758	7.89	220	8.01	64	8.63	1	6.67
Intermenstrual bleeding	1545	16.07	440	16.01	101	13.61	2	13.33
Age (years)*								
Median	36	29.0, 42.0)	36	(30.0, 43.0)	38	(31.0, 44.0)	43	(35.5, 48.2)
< 20	235	2.44	37	1.35	13	1.75	0	0.00
20 ~ 29	1057	11.00	564	20.52	120	16.17	0	0.00
30 ~ 39	1877	19.53	1059	38.54	235	31.67	5	33.33
40 ~ 49	1422	14.79	749	27.26	227	30.59	8	53.33
≥ 50	288	3.00	211	7.68	60	8.09	1	6.67
Unknow	4734	49.25	128	4.66	87	11.73	1	6.67
Dose**								
Dose 1	4966	51.66	1388	50.51	465	62.67	9	60.00
Dose 2	3563	37.06	923	33.59	1	0.13	2	13.33
Dose 3	65	0.68	17	0.62	0	0.00	0	0.00
Unknown	1019	10.60	420	15.28	276	37.20	4	26.67
*Serious adverse event*								
Life-threatening	66	0.69	9	0.33	6	0.81	0	
Hospitalized	238	2.48	46	1.67	12	1.62	0	
Prolonged hospitalization	0		1	0.04	1	0.13	0	
Disability	550	5.72	117	4.26	20	2.70	1	6.67
*History and current*								
Taking medications	3441	35.80	1398	50.87	406	54.72	8	53.33
Current Diseases	587	6.11	327	11.90	68	9.16	2	13.33
Past Diseases	4874	50.70	1046	38.06	277	37.33	7	46.67
*Recovrd*								
No	6376	66.33	1465	53.31	401	54.04	7	46.67
Yes	1596	16.60	628	22.85	136	18.33	3	20.00
Unknow	1424	14.81	521	18.96	165	22.24	5	33.33

Multiple MedRDA terms can exist in one adverse reaction report, so the sum of the individual events ofmenstrual disorders events may be greater than the total number of reports

Fisher’s exact test: α = 0.05,

*P value < 0.001

**P value < 0.001

### Reporting odds ratio

Analyses of the stated odds ratio for the COVID-19 vaccination incidents are shown in Tables 3, 4, 5, 6. The distribution of adverse events according to type (Menstrual disorder vs. other adverse reactions) and vaccination status (COVID-19 vaccines vs. other vaccines) is reported in Table 3. ROR estimated to be 7.83 (95% CI: 7.39–8.28), implies that COVID-19 vaccine may be a risk sign for the occurrence of events related to menstrual disorders. To further validate the correlation, three sensitivity analysis of the ROR were also performed. Firstly, aggregated by region of adverse reaction reporting (US vs. non-US) and vaccination status, ROR was 0.78(95% CI: 0.70–0.88), suggested that the reports of menstrual disorders after vaccination with the COVID-19 vaccine are unrelated to the regional distribution. Secondly grouped by age and type of report, compared the reported rates of adverse events associated with menstrual disorders in the 30–49 age group with those in other age groups, ROR was 5.78(95% CI: 4.86–6.88). Finally, excluding reports of unknown age, ROR was 12.46(95% CI: 10.41–14.92). Suggests that age may be a risk indicator for the event of menstrual disorders after vaccination with the COVID-19 vaccine.

**Table 3:** Distribution of adverse events stratified by menstrual disorders events and vaccination status

	Menstrual disorders events	Other adverse events	Total
COVID-19 vaccine	13,118	587,325	600,443
Other vaccines	1313	460,130	461,443
Total	14,431	1,047,452	1,061,886

ROR = 7.83, (95%CI: 7.39–8.28)

**Table 4:** Sensitivity analyses1 (Distribution of adverse events stratified by region and vaccination status)

	U.S	Non-U.S	Total
COVID-19 vaccine*	6431	6687	13,118
Other vaccines**	724	589	1313
Total	7155	7276	14,431

*Menstrual disorders events reported after exposure to COVID-19 vaccine

**Menstrual disorders events reported after exposure to other vaccines

ROR = 0.78, (95%CI:0.70–0.88)

**Table 5:** Sensitivity analyses2 (Distribution of adverse events stratified by age and vaccination status)

	30–49 ages	Other ages	Total
COVID-19 vaccine*	5582	7536	13,118
Other vaccines**	149	1164	1313
Total	5731	8700	14,431

*Menstrual disorders events reported after exposure to COVID-19 vaccine

**Menstrual disorders events reported after exposure to other vaccines

ROR = 5.78, (95%CI: 4.86–6.88)

**Table 6:** Sensitivity analyses3 (Distribution of adverse events stratified by age and vaccination status after exclusion of unknown age)

	30–49 ages	Other ages	Total
COVID-19 vaccine*	5582	2586	8168
Other vaccines**	149	860	1009
Total	5731	3446	9177

*Menstrual disorders events reported after exposure to COVID-19 vaccine

**Menstrual disorders events reported after exposure to other vaccines

ROR = 12.46, (95%C: 10.41–14.92)

## Discussion

Coronavirus disease 2019 (COVID-19) is as an emerging infectious disease (EID) responsible for the worst public health catastrophe of the twenty-first century thus far. In order to contain the spread of the epidemic, countries around the world have launched vaccination campaigns for COVID-19 vaccine and have adopted various measures to monitor for adverse reactions after vaccination. VAERS as a vaccine adverse reaction self-reporting system, a total of 14,431 menstrual disorder-like events were reported by female in VAERS, and 13,118 (90.90%) were exposed to the COVID-19 vaccine, from the inception of the database in 1990 to November 12, 2021. The general characteristics of the reports showed that female in the age group 30–49 years reported the highest number of incidents of menstrual disorders compared to other vaccines. The highest proportion of reports was for the Pfizer-Biontech vaccine, with 1065 reports mentioning serious adverse events. The majority of reports of menstrual disorders occurred after the first dose of the COVID-19 vaccine, with only about 0.5% reported after the third dose of the vaccine. The interval between vaccination and adverse reaction reporting was within 100 days in 82.92% of patients, but only 18.01% of patients reported that their adverse reactions had vanished, and 62.88% of patients reported that they still had adverse reactions related to vaccination.

To my knowledge, this is the first time that a menstrual disorder-related incident has been correlated to the COVID-19 vaccine. Normal female menstruation is judged by the cycle of menstruation, the period, the volume, the color, the quality and the concomitant symptoms of menstruation. Among the events of menstrual disorders reported by the COVID-19 vaccine, Menstruation irregular was found to be the most frequently reported, followed by Menstruation prolonged, Intermenstrual bleeding and Menstrual disorder, with Menorrhagia being the least frequently reported. Dietary nutrition, environmental factors, lifestyle and mental health conditions are important factors affecting menstrual irregularities in female of reproductive age [24]. During the covid-19 epidemic, female were more vulnerable to stress and were substantially more depressed and agitated than men [25], and middle-aged females are the major groups for the first vaccination. This may explain the climb in reports of menstrual disorders after vaccination with the COVID-19 vaccine. Our study found a potential association signal between COVID-19 vaccine and menstrual disorders occurrences with ROR = 7.83 and fulfilled the lower criterion of ROR 95% CI > 1 and number of cases > 3. Conversely, adverse reactions were reported independent of region, with ROR < 1. In addition, studies [26] have ascertained a greater correlation between prolonged and irregular menstrual cycles and the risk of premature death (age < 70 years), highlighting that menstrual disorders in female must be handled seriously enough.

Vaccination with COVID-19 causes adverse reactions in adults differently depending on their age and gender. However, after the launch of COVID-19 vaccine, attention to adverse reactions associated with vaccination has been concentrated on the elderly [8], adolescents [9], and even pregnant female and young children [27], while attention to young adult female has been lacking. However, female of reproductive age account for the majority of adverse reaction reports received by VAERS for menstrual disorders. Our study found that reports of adverse reactions to menstrual disorders in females aged 30–49 years increased nearly tenfold in comparsion with the pre-marketing period of the vaccine. There was a danger signal between this age group of female compared to other age groups for menstrual disorders events after COVID-19 vaccination with a lower limit of the ROR 95% CI > 1. To overcome some limitations of the spontaneous data, such as missing data in the report, a sensitivity analysis was conducted to exclude age unknowns and produce comparable results. Reports of menstrual disorder-like events after vaccination of female with the COVID-19 vaccine are age-related, and females of reproductive age between 30 and 49 years old need to pay particular attention to adverse reactions after vaccination.

Three COVID-19 vaccines are authorized or approved for use among adults in the United States, respectively: mRNA-1273 from Moderna [5], BNT162b2 from Pfizer-BioNTech [4], Ad26.COV2 from Janssen [6]. Inoculation with Pfizer-BioNTech accounted for the majority of reports of menstrual disorders, with Moderna in second place and Janssen in third. As of November 2, 2021, approximately 248 million doses of the Pfizer-BioNTech COVID-19 vaccine had been administered to persons aged ≥ 12 years in the United States [28]. And as of November 12, 2021, VAERS has received reports of menstrual disorders representing approximately 1 in 100,000 of the total number of Pfizer vaccinations. Adverse reactions to vaccination appear to be negligible compared to the serious life-threatening repercussions of infection with the virus, and there is a lack of much substantial evidence linking vaccination to adverse reactions [29, 30]. Bell’s palsy [31] (or Acute unilateral facial nerve palsy) has been observed as a uncommon neurological adverse effect in preliminary clinical studies with COVID-19 vaccine. Similarly, Eric Wan and colleagues in *The Lancet Infectious Diseases* demonstrated the overall increased risk of Bell's palsy following COVID-19 vaccination. The COVID-19 vaccine, however, appears not to be linked to Bell's palsy until further research is conducted [32]. Based on various studies of real-world data [33, 34], all approved or licensed COVID-19 vaccines provide substantial protection, although the degree of protection varies among vaccines.

Nevertheless, there are several limitations to our study. First, it’s not abundantly clear whether these adverse reactions were caused by disease or exposure to the vaccine, which is a major drawback of our research. It also not feasible to rule out the inclusion of alterations induced by COVID-19 infection, and there is a paucity of data related to specific medical history and menstrual histories. Second, since VAERS is dependent on spontaneous reporting, our results are susceptible to numerous biases, such as data omission, overreporting, and underreporting. Third, co-vaccination may suppress or augment the immune response, and this study solely pertains to the report of vaccination alone and does not include the data of co-vaccination. There is a possibility that our results may be influenced by all of the above.

## Conclusion

From the signal detection results, there was a statistically significant correlation between the COVID-19 vaccine and reports of adverse reactions to menstrual disorders, and COVID-19 may contribute to menstrual disorders in young adult female (30–49 years).However, our study data are from VAERS and the results may be influenced by the number and quality of reports, so it is challenging to study menstrual disorders plainly categorized as COVID-19 vaccine adverse events. It should continue to track adverse reactions to vaccine in female in this age group.

## Data Availability

We sincerely thank the VAERS for providing research data. This data can be found at: https://vaers.hhs.gov/data/datasets.html.
